# The impact of the COVID‐19 pandemic on the diagnosis and management of pre‐eclampsia: Identifying healthcare delays

**DOI:** 10.1002/ijgo.70434

**Published:** 2025-08-09

**Authors:** Juliana da‐Costa‐Santos, Vitor V. L. Reis, Rodolfo R. Japecanga, Mariana Seabra, Maykon Anderson Carvalho, Ana Julia Ganzeli Oliveira, Laura Cintra Vinchi, Arthur B. P. Righi, Jussara Mayrink, José Paulo S. Guida, Renato T. Souza, José G. Cecatti, Maria Laura Costa

**Affiliations:** ^1^ Department of Obstetrics and Gynecology, School of Medical Sciences Universidade Estadual de Campinas Campinas Brazil; ^2^ Pontifical Catholic University of Campinas Brazil; ^3^ Department of Obstetrics and Gynecology, School of Medicine Federal University of Minas Gerais Belo Horizonte Brazil

**Keywords:** COVID‐19, pandemic preparedness, pre‐eclampsia, pregnancy complications, public health surveillance

## Abstract

**Objective:**

To compare the prevalence of pre‐eclampsia with and without severe features, and maternal and perinatal outcomes before and during the COVID‐19 pandemic.

**Methods:**

Cross‐sectional study based on medical chart review of pregnant women admitted to a referral maternity hospital for childbirth between September 2019 to February 2020 (before the pandemic) and March 2020 to August 2021 (during the pandemic, subdivided into three 6‐month periods). The prevalence of pre‐eclampsia, maternal, and perinatal outcomes were compared.

**Results:**

A total of 3914 deliveries were considered, 1059 pre‐pandemic and 2855 during the pandemic. The overall prevalence of pre‐eclampsia was 10.9%, not differing significantly among periods (9.3%–14.3%, *P* = 0.144). Social and demographic characteristics were similar between groups. The use of anti‐hypertensive therapy during pregnancy decreased significantly (69.9% pre‐pandemic versus 47.1%–54.0% during the pandemic, *P* = 0.007). The diagnosis of early‐onset pre‐eclampsia reduced significantly (37.9% before and 19.3%–30.2% during the pandemic, *P* = 0.025), and so did the proportion of severe pre‐eclampsia (61.1% before and 38.1%–58.3% during the pandemic, *P* = 0.001). However, the gestational age at birth was similar between groups, around 36 weeks. COVID‐19 positivity increased from 0.9% to 12.0% during the pandemic periods (*P* < 0.001). There were no maternal deaths. Newborns were less often admitted to intensive care during the pandemic, and over 60% of these admissions were the result of prematurity in all periods studied. Other perinatal outcomes remained similar.

**Conclusion:**

The prevalence of pre‐eclampsia increased during the study period, with diagnoses occurring at later gestational ages, likely due to delays in healthcare access.

## 1 INTRODUCTION

The COVID‐19 pandemic has significantly impacted health care globally, especially in low‐ and middle‐income country settings. During the first few months, before vaccination, there was a marked reduction in the number of outpatient consultations, to prioritize the care of those with suspected infection, and to avoid risks of contamination in health facilities.^1^, ^2^, ^3^ Settings with restrictions to implement telehealth canceled and delayed visits, leading to insufficient counseling.^4^ In Obstetrics, prenatal care is key for the follow up during pregnancy, and delays in such intervention could impact the timely identification of obstetric risks and complications.^5^ Among the main complications, pre‐eclampsia stands out as one of the most relevant causes of maternal morbidity and mortality.

Previous studies suggest a higher frequency of pre‐eclampsia in pregnant individuals with COVID‐19, potentially due to shared pathophysiology involving the angiotensin‐converting enzyme II (ACE‐2) receptor, which has a role in blood pressure regulation through the renin‐angiotensin pathway.^6^, ^7^ SARS‐CoV‐2 enters cells via this receptor, which is highly expressed in the placenta during the third trimester, potentially reducing its availability.^8^, ^9^, ^10^ In fact, lower levels of this protein have been associated with pre‐eclampsia.^11^

There is also evidence of adverse maternal outcomes in cases of concurrent diagnosis of COVID‐19 and pre‐eclampsia, considering that the conditions share some risk factors for severe disease.^6^ Before vaccination, a challenge for the differential diagnosis between HELLP (hemolysis, elevated liver enzymes and low platelets) syndrome and severe COVID‐19 was reported, as both conditions can present multi‐organ damage, with great inflammatory response and vascular activation.^12^

Hence, considering the direct and indirect effects of the COVID‐19 pandemic and the possible reduction in the prevention, diagnosis, and treatment of hypertensive disorders in pregnancy, this study aims to compare the prevalence of pre‐eclampsia with and without signs of severity and maternal and perinatal outcomes before and during the pandemic in a referral center in Brazil, during a 2‐year interval, contrasting the 6 months before the beginning of the pandemic and 18 months during the pandemic.

## 2 | MATERIALS AND METHODS

This retrospective cross‐sectional study used a convenience sample. After reviewing medical charts of pregnant women admitted to the Women's Hospital of the University of Campinas for childbirth, we selected those who had any combination of criteria for pre‐eclampsia according to the International Society for the Study of Hypertension in Pregnancy guidelines (ISSHP) guidelines^13^: hypertension with proteinuria, or hypertension with signs of end‐organ damage, such as neurologic symptoms, upper‐left quadrant abdominal pain, and altered laboratory workup.

This maternity hospital is responsible for the Obstetric tertiary care of a region with more than 3 000 000 inhabitants. Childbirth was defined as delivery after at least 20 weeks of pregnancy. Cases were screened from September 1, 2019, to August 31, 2021. The 2‐year period was divided into four shorter intervals: before the pandemic (September 2019 to February 2020), and three pandemic periods (March to August 2020, September 2020 to February 2021, and March to August 2021).

The first period ended in February 2020 because the first case of COVID‐19 in the country was confirmed on February 26, 2020.^14^ The second period began in March, supported by the introduction of the disease in Brazil and the declaration of the pandemic by the World Health Organization,^15^ and ended in August 2020. At that time, although the numbers of infected people were very high, there was a clear deceleration of the contagion both in Brazil (to a third of the numbers before this period) and, for the first time, a deceleration happened in the region of the city of Campinas.^16^, ^17^ From September 2020 to February 2021, there was marked acceleration of contamination in the country and the region of Campinas. This reached its peak by mid‐January, quickly decreasing to levels similar to September 2020 in February in Campinas,^18^ hence ending the third period of data collection. In Brazil and in Campinas, March 2021 was the month the people were most affected by COVID,^16^, ^18^ supporting the beginning of the last period of data. As all the previous periods were for 6 months, for symmetry, the fourth period ended in August 2021.

**FIGURE 1:**
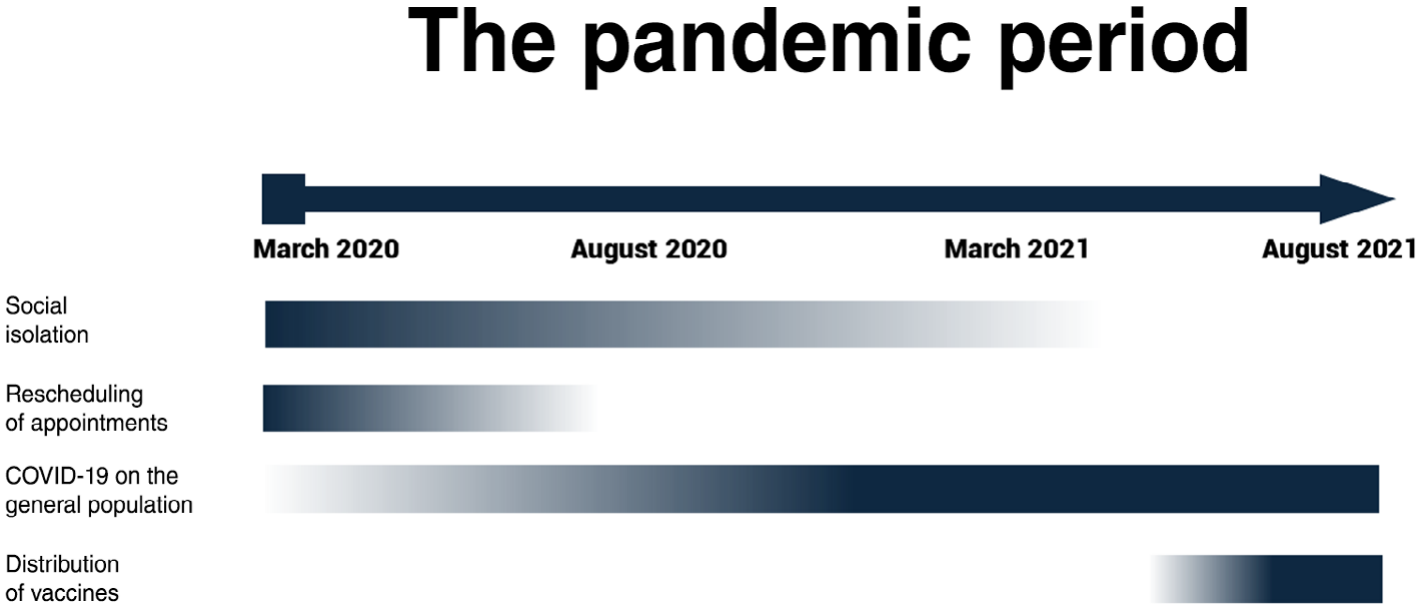
Pandemic subdivisions considered for the present analysis. The color intensity of social isolation, rescheduling of appointments, effect of COVID‐19 on the general population, and distribution of vaccines reflects how much each factor interfered with health care at the time points March 2020, August 2020, March 2021, and August 2021. Social isolation decreased progressively, whereas the impact of COVID‐19 on the general population intensified. Rescheduling of medical appointments was restricted to the first months of the pandemic, and the distribution of vaccines began in May 2021.

The subdivision of the pandemic is also justified by the different impacts it had: the first 6 months were marked by social isolation and rescheduling of appointments; from the 7th month to a year into the pandemic there was a heavy impact of COVID‐19 on the general population and also social isolation, and from the 13th month to 1.5 years of the pandemic there was the beginning of the distribution of vaccines. Figure 1 schematically displays the subdivision of the pandemic periods.

Pre‐eclampsia was defined according to the ISSHP if the pregnant woman presented, at or after 20 weeks of pregnancy, with hypertension and any of the following clinical or laboratory end‐organ injuries, such as proteinuria, HELLP syndrome, acute renal injury, acute pulmonary edema, or visual or neurologic symptoms.^13^ We also considered placental insufficiency (such as fetal growth restriction and/or oligohydramnios) diagnosed by ultrasound as a criterion for pre‐eclampsia. Pre‐eclampsia with signs of severity was considered when there was any combination of hypertension and/or symptoms and/or altered laboratory findings, or magnesium sulfate was indicated to prevent seizures, according to the ISSHP.^13^ Superimposed pre‐eclampsia was considered when pre‐eclampsia was diagnosed in a woman with a previous diagnosis of chronic hypertension (before pregnancy or during pregnancy, but before the 20 weeks of pregnancy).

The prevalence of pre‐eclampsia and its severe features before and during the pandemic were compared. Maternal information about social and demographic characteristics, obstetric history, use of antihypertensive drugs, use of magnesium sulfate from pregnancy to the postpartum period, labor and delivery descriptions, and perinatal outcomes were also examined and compared among groups. Labor induction was considered successful if a vaginal birth occurred following induction of labor.

For the analyses, descriptive statistics involved qualitative variables in frequencies and percentages, and quantitative variables were described as means ± standard deviations. The four 6‐month intervals (before and during the pandemic) were compared. The association of categorical variables was described with the *χ* ^2^ or Fisher exact test, whereas numerical ones were associated with the *t* test or Mann–Whitney *U* test, depending on the distribution of the data. Analyses were performed with software R, version 4.3.0, and *P* values below 0.05 were considered significant. The current report follows the STROBE guidelines.^19^

The study was approved by the local Ethical Review Board, under the reference number #60249222.3.0000.504. Data confidentiality was assured, and informed consent was waived because of the retrospective nature of the study and the fact that women do not proceed to long‐term follow up in Obstetrics at the institution after childbirth. The Ethical Review Board, however, required a term for the use of data that took place to ascertain the safety and anonymity of information.

## 3 | RESULTS

**FIGURE 2:**
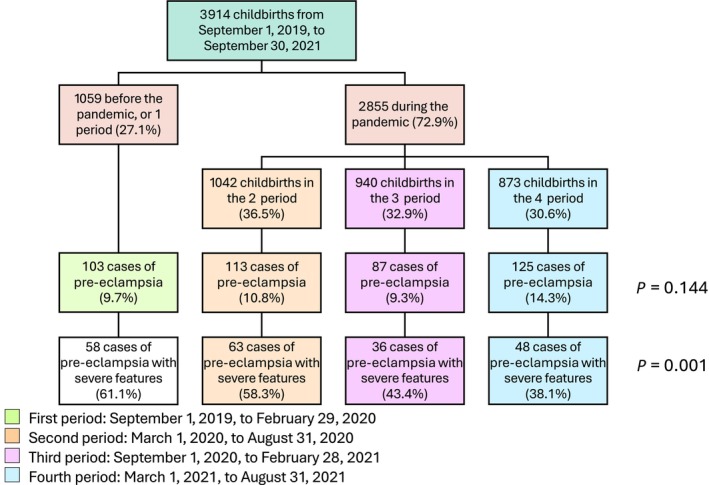
Flow chart of patient selection. Severity was considered if magnesium sulfate was needed for severe features of pre‐eclampsia (neuroprotection not included) or if there was any other outcome indicative of severity, such as HELLP (hemolysis, elevated liver enzymes and low platelets) syndrome, eclampsia, or placental abruption.

During the study period, 3914 women delivered in the referral maternity hospital and their medical charts were screened for the diagnosis of pre‐eclampsia. The women were divided into four subgroups according to the timing of childbirth: the first subgroup had 1059 women from September 1, 2019, to February 29, 2020, the second had 1042 women from March 1, 2020, to August 31, 2020, the third had 940 women from September 1, 2020, to February 28, 2021, and the fourth had 873 women from March 1 until August 31, 2021. Pre‐eclampsia was diagnosed in 428 women (overall prevalence of 10.9%). There was no significant difference in the prevalence of pre‐eclampsia, which was the lowest in the third period (9.3%) and peaked at 14.3% during the last period (*P =* 0.144). Figure 2 shows the flow chart of patient selection.

The social and demographic characteristics that were similar throughout the study were age around 30 years old and schooling up to high school. There was a significant difference regarding skin color, paid work in pregnancy, marital status, parity, obesity, and diabetes: the third period showed the highest proportion of Pardo, working, with partner, diabetic, and obese women, and the smallest number of primigravida (Table 1).

**TABLE 1:** Social and demographic characteristics, clinical and obstetric history among women with preeclampsia before and during the COVID‐19 pandemic.^a^

Characteristics	Pre‐eclampsia before the pandemic	Pre‐eclampsia during the pandemic			*P*‐value
	September 2019 to February 2020	March to August 2020	September 2020 to February 2021	March to August 2021	
Cases of pre‐eclampsia	103 (9.7%)	113 (10.8%)	87 (9.3%)	125 (14.3%)	0.144
Maternal age, years	30.2 ± 7.3	29.7 ± 6.8	30.7 ± 6.5	30.8 ± 7.0	0.430
Skin color^b^					
White	68 (67.3%)	67 (60.9%)	42 (48.3%)	89 (73.0%)	0.004
Pardo	22 (21.8%)	35 (31.8%)	38 (43.7%)	24 (19.7%)	
Black	11 (10.9%)	8 (7.3%)	7 (8.0%)	9 (7.4%)	
Paid work in pregnancy^c^	62 (62.0%)	64 (61.5%)	67 (77.9%)	73 (61.3%)	0.047
Marital status with partner	73 (70.9%)	72 (63.7%)	71 (81.6%)	86 (68.8%)	0.049
Schooling^d^					
Up to High school	80 (81.6%)	94 (83.9%)	80 (92.0%)	100 (80.6%)	0.134
College or Postgraduation	18 (18.4%)	18 (16.1%)	7 (8.0%)	24 (19.4%)	
Primigravida	36 (35.0%)	47 (41.6%)	20 (23.0%)	46 (36.8%)	0.048
Obesity^e^	27 (71.1%)	69 (81.2%)	60 (82.2%)	73 (67.0%)	0.049
Diabetes^f^	33 (32.0%)	42 (37.2%)	45 (51.7%)	48 (38.7%)	0.043
Chronic hypertension	38 (36.9%)	32 (28.3%)	38 (43.7%)	30 (24.0%)	0.012
Use of anti‐hypertensive drugs in pregnancy	72 (69.9%)	61 (54.0%)	41 (47.1%)	65 (52.0%)	0.007
Classes of anti‐hypertensive drugs used					
1	55 (76.4%)	52 (85.2%)	40 (95.2%)	55 (83.3%)	0.008
2	14 (19.4%)	5 (8.2%)	2 (4.8%)	9 (13.6%)	
3	3 (4.2%)	4 (6.6%)	0 (0%)	2 (3.0%)	
Confirmed COVID‐19 during pregnancy^g^	Not applicable	1 (0.9%)	4 (4.6%)	15 (12.0%)	<0.001
Tobacco smoking	7 (6.8%)	6 (5.3%)	8 (9.2%)	101 (8.0%)	0.738
Alcohol consumption	7 (6.8%)	1 (0.9%)	10 (11.5%)	3 (2.4%)	0.002
Substance abuse	4 (3.9%)	1 (0.9%)	3 (3.4%)	7 (5.6%)	0.232

^a^ Data are presented as mean ± standard deviation or as number (percentage).

^b–f^ Missing values: ^b^8; ^c^19; ^d^7; ^e^123; ^f^1.

^g^ Confirmed COVID‐19 was considered when there was a documented positive molecular test.

There was a significant difference between the rates of superimposed pre‐eclampsia, before and during the pandemic (36.9% of chronic hypertension before versus 28.3%–43.7% during the pandemic, *P* = 0.012). The proportion of women with pre‐eclampsia using antihypertensive drugs was significantly different before (70.9%) and during (47.1%–54.0%) the pandemic (*P* = 0.007). Monotherapy was significantly more prevalent during the pandemic (76.4% before and 83.3%–95.2% during the pandemic, *P* = 0.008). The diagnosis of diabetes was significantly less frequent before (32.0%) than during the pandemic (37.2%–51.7%, *P* = 0.043). The prevalence of confirmed COVID‐19 via molecular testing increased significantly during the pandemic (from 0.9% to 12.0%, *P* < 0.001). Apart from alcohol consumption (6.8% before and 0.9%–11.5% during the pandemic, *P* = 0.002), smoking and the abuse of other substances were similar between groups. Further detail on the social, demographic, obstetric and clinical characteristics of enrolled women are shown in Table 1.

The diagnosis of pre‐eclampsia and its signs of severity are shown in Table 2. Over 90% of the pregnancies were singleton (*P* = 0.825), and the diagnosis of pre‐eclampsia was established before or at childbirth, with similar proportions before and during the pandemic (*P* = 0.353). Even though most diagnoses were made during pregnancy, there was a significant reduction in the diagnosis of early‐onset pre‐eclampsia (diagnosed before 34 weeks of pregnancy): before the pandemic, 37.9% of pre‐eclampsia was early‐onset, while during the pandemic this percentage ranged from 19.3% to 30.2% (*P* = 0.025). Among these early‐onset cases, corticosteroids for lung maturation were significantly more frequently used before than during the pandemic (33.0% before and 0%–20.0% during the pandemic, *P* = 0.010). On the other hand, the proportion of diagnoses at term increased to 51.8% during the pandemic, compared with 32.6% before the pandemic (*P* = 0.018). The difference between the mean time from the diagnosis of pre‐eclampsia to severity before (2.14 ± 7.1 days) and during the pandemic (1.19 ± 3.8 to 2.97 ± 12.8 days, *P* = 0.658) was not significant. Likewise, the mean gestational age at childbirth was similar before (35.2 ± 3.9 weeks) and during the pandemic (36.0 ± 2.9 to 36.4 ± 2.5 weeks, *P* = 0.185).

**TABLE 2:** Diagnosis of pre‐eclampsia and severe preeclampsia before and during the COVID‐19 pandemic.^a^

Diagnosis of pre‐eclampsia	Pre‐eclampsia before the pandemic	Pre‐eclampsia during the pandemic			*P*‐value
	September 2019 to February 2020	March to August 2020	September 2020 to February 2021	March to August 2021	
Type of pregnancy					
Singleton	95 (92.2%)	106 (93.8%)	83 (95.4%)	116 (92.8%)	0.825
Multiple^b^	8 (7.8%)	7 (6.2%)	4 (4.6%)	9 (7.2%)	
Moment of diagnosis of PE					
Pregnancy^c^	95 (92.2%)	108 (95.6%)	83 (95.4%)	126 (90.6%)	0.353
Postpartum	8 (7.8%)	5 (4.4%)	4 (4.6%)	13 (9.4%)	
Early‐onset PE^d^					
No	59 (62.1%)	83 (76.9%)	67 (80.7%)	88 (69.8%)	0.025
Yes	36 (37.9%)	25 (23.1%)	16 (19.3%)	38 (30.2%)	
Corticosteroids for fetal lung maturation among early‐onset PE^d^	12 (33.3%)	5 (20.0%)	0 (0%)	3 (7.9%)	0.010
PE diagnosed at term^e^	31 (32.6%)	46 (42.6%)	43 (51.8%)	52 (41.3%)	0.018
Severe PE^e^	58 (61.1%)	63 (58.3%)	36 (43.4%)	48 (38.1%)	0.001
GA at first severe PE,^e^ weeks	32.7 ± 4.4	34.9 ± 3.1	34.6 ± 3.7	34.1 ± 3.3	0.025
Time between the diagnosis of PE and severity,^d^ days	2.14 ± 7.1	1.19 ± 3.8	2.97 ± 12.8	2.35 ± 8.1	0.658
GA at childbirth,^e^, ^f^ weeks	35.2 ± 3.9	36.1 ± 2.8	36.4 ± 2.5	36.0 ± 2.9	0.185
Eclampsia	2 (1.9%)	2 (1.8%)	0 (0%)	2 (1.6%)	0.671

Abbreviations: GA, gestational age; PE, pre‐eclampsia.

^a^ Data are presented as mean ± standard deviation or number (percentage).

^b^ Pregnancies with triplets: 2.

^c^ 17 intrapartum diagnoses.

^d^ Only early‐onset pre‐eclampsia was considered (*n* = 115).

^e^ Only pre‐eclampsia diagnosed during pregnancy was considered (*n* = 412).

^f^ Missing data: 1.

Regarding pre‐eclampsia with signs of severity, 61.1% of the cases of the first period (before the pandemic) were severe, with gradual decrease in percentage during the pandemic, reaching its lowest in the fourth period (38.1%, *P* = 0.001). Among those who developed severe features during pregnancy or childbirth, the mean GA of diagnosis was significantly different: 32.7 ± 4.4 weeks before the pandemic versus 34.1 ± 3.3 weeks to 34.9 ± 3.1 weeks during the pandemic (*P* = 0.025).

Regarding hospitalization for childbirth, few women were admitted in spontaneous labor and similarly among groups (pre‐pandemic: 12.6% and pandemic: 7.1%–14.4%, *P* = 0.281). Excluding those who were admitted in labor, women who had placental abruption, and women with two or more previous cesarean sections, induction of labor was significantly more frequent during the pandemic (before: 43.7% and during the pandemic: 51.6%–72.5%, *P* = 0.001). However, labor induction succeeded similarly: 48.4% before and 38.3%–59.2% during the pandemic (*P* = 0.130). Cesarean sections as the final route of delivery were predominant, with 74.8% before and 69.6%–76.1% during the pandemic (*P* = 0.690). At discharge, women were under anti‐hypertensive therapy in 70.9% of the cases before the pandemic, and from 51.2% to 66.4% of the cases during the pandemic (*P* = 0.011). There were no maternal deaths in the period studied.

Most of the perinatal outcomes were similar throughout the period studied: fetal deaths ranged from 0% to 2.7% (*P* = 0.314), neonatal deaths ranged from 1.1% to 4.5% (*P* = 0.545), 5‐min Apgar score below 7 occurred in 3.3%–7.5% of the newborns (*P* = 0.522), and the mean birth weight was 2540–2840 g (*P* = 0.143). The frequency of small‐for‐gestational‐age babies remained stable (19.6% before and 17.6%–19.4% during the pandemic), but the proportion of large‐for‐gestational‐age (LGA) was significantly higher during the pandemic (4.7% before and 9.7%–20.9% during the pandemic, *P* = 0.032). Before the pandemic, the frequency of admissions at the neonatal intensive care unit (ICU) was significantly higher than during the pandemic (54.2% versus 37.3%–42.0%, *P* = 0.037). The main reasons for admission were prematurity (67.2% before versus 64.7%–86.0% during the pandemic, *P* = 0.077), low birth weight (56.9% before and 60.0%–64.0%, *P* = 0.846), and respiratory support (62.1% before the pandemic and 64.0%–74.0% during the pandemic, *P* = 0.477) (Table 3).

**TABLE 3:** Hospitalization for childbirth and maternal and perinatal results among women with pre‐eclampsia before and during the COVID‐19 pandemic.^a^

Maternal and perinatal outcomes	Pre‐eclampsia before the pandemic	Pre‐eclampsia during the pandemic			*P*‐value
	September 2019 to February 2020	March to August 2020	September 2020 to February 2021	March to August 2021	
Spontaneous labor	13 (12.6%)	8 (7.1%)	8 (9.2%)	18 (14.4%)	0.281
Labor induction^b^	31 (43.7%)	60 (64.5%)	50 (72.5%)	49 (51.6%)	**0.001**
Success of labor induction^c^	15 (48.4%)	23 (38.3%)	20 (40.0%)	29 (59.2%)	0.130
Final route of delivery					
Vaginal	26 (25.2%)	27 (23.9%)	24 (27.6%)	38 (30.4%)	0.690
Cesarean section	77 (74.8%)	86 (76.1%)	63 (72.4%)	87 (69.6%)	
Fetal death^d^	3 (2.7%)	3 (2.5%)	0 (0%)	1 (0.7%)	0.314
Neonatal death^e^	2 (1.9%)	2 (1.7%)	1 (1.1%)	6 (4.5%)	0.435
Birth weight,^e^ g	2540 ± 882	2720 ± 743	2840 ± 832	2630 ± 833	0.143
Adequacy of birthweight/GA^e^					
SGA	21 (19.6%)	21 (17.6%)	17 (18.7%)	26 (19.4%)	0.032
AGA	81 (75.7%)	84 (70.6%)	55 (60.4%)	95 (70.9%)	
LGA	5 (4.7%)	14 (11.8%)	19 (20.9%)	13 (9.7%)	
5‐min Apgar score <7^e^	8 (7.5%)	6 (5.0%)	3 (3.3%)	5 (3.7%)	0.522
Admission to the neonatal ICU^e^	58 (54.2%)	50 (42.0%)	34 (37.4%)	50 (37.3%)	0.037
Main reasons for admission at the neonatal ICU^f^					
Prematurity	39 (67.2%)	33 (66.0%)	22 (64.7%)	43 (86.0%)	0.077
Low birth weight	33 (56.9%)	30 (60.0%)	21 (61.8%)	32 (64.0%)	0.846
Respiratory support	36 (62.1%)	32 (64.0%)	22 (64.7%)	37 (74.0%)	0.477
Anti‐hypertensive therapy at maternal discharge	73 (70.9%)	75 (66.4%)	57 (65.5%)	64 (51.2%)	0.011

Abbreviations: AGA, adequate for gestational age; GA, gestational age; ICU: intensive care unit; LGA: large for gestational age; SGA, small for gestational age.

^a^ Data are presented as mean ± standard deviation or number (percentage).

^b^ Labor induction only considers those who did not have spontaneous labor or placental abruption, among women with none or one previous cesarean section (*n* = 328).

^c^ Vaginal birth after labor induction only considers those who underwent labor induction (*n* = 190).

^d^ Total of fetuses analyzed, *n* = 458.

^e^ Excludes fetal deaths (number considered = 451).

^f^ Excludes newborns who were not admitted to the neonatal intensive care unit (number considered = 192).

## 4 | DISCUSSION

This study did not show a significant difference in the prevalence of pre‐eclampsia, from around 10% pre‐pandemic and during the first year of the pandemic to 14.3% from March to August 2021. Although the latter had the highest prevalence of pre‐eclampsia, it had the lowest proportion of pre‐eclampsia with severe features and women with chronic hypertension (hence, superimposed pre‐eclampsia). During the pandemic, there was a significant reduction in the detection of early‐onset pre‐eclampsia in parallel with an increase in detection of pre‐eclampsia at term. There was also a reduction in anti‐hypertensive therapy use during pregnancy and at discharge during the pandemic. The frequency of confirmed maternal infections with COVID‐19 increased during the period studied. When admitted for delivery, women underwent induction of labor more often during the pandemic, even though cesarean sections remained stable, with rates around 70%. The prevalence of the diagnosis of maternal diabetes and LGA newborns fluctuated similarly throughout the period studied: when the proportion of diabetic women increased, the proportion of LGA also increased. Apart from fewer neonatal ICU admissions during the pandemic, there were no differences in other perinatal outcomes.

In many low‐ and middle‐income settings, pre‐eclampsia is the main cause of maternal mortality and morbidity. For instance, a systematic analysis from the World Health Organization from 2009 to 2020 has found that 22% of all maternal deaths in Latin America and the Caribbean were due to hypertensive disease, which corresponds to over 21 000 women. In contrast, 12% of the maternal deaths in Europe and North America were due to hypertension in the same period, which corresponds to around 2000 women.^20^ In Brazil, despite improvements in general maternal and perinatal health indicators before the pandemic, numbers related to hypertension did not decrease: the estimated prevalence of pre‐eclampsia in the country is 6.7%,^21^ and pre‐eclampsia is responsible for around 25% of the maternal deaths, accounting for around 320 maternal deaths per year in the last decade.^22^

The current study showed a high prevalence of pre‐eclampsia in all periods, most likely because of the high‐risk background of the women assisted at the referral center considered, which was particularly important during the pandemic because there were administrative restrictions on hospitalizing patients who were not part of the hospital's prenatal outpatient clinic who might have low‐risk pregnancies. This high prevalence follows other studies that report increasing numbers of women with hypertension during pregnancy, as a consequence of multiple factors such as obesity, increased maternal age, and underlying conditions like chronic hypertension and diabetes.^23^, ^24^ Moreover, the increasing figures for pre‐eclampsia can be demonstrated within the institution, as a previous study from the same referral hospital in 2017 reported a prevalence of pre‐eclampsia of 7.3%.^25^

This study showed a significant decrease in the diagnosis of pre‐eclampsia with severe features and fewer diagnoses of early‐onset pre‐eclampsia, which is known to be more severe than late‐onset pre‐eclampsia. Contrastingly, data from the Brazilian Public Health System indicate that severe maternal morbidity owing to hypertension during the pandemic was higher than the national average in the region this maternity hospital encompasses. From 2018 to 2019, national and regional figures of severe maternal morbidity due to hypertension were around 52%.^26^ From 2020 to 2021, Brazil had 53.6% and the region of Campinas had 56.4% of severe maternal morbidity related to hypertension.^26^

There are suggested mechanisms of a shared pathophysiology between pre‐eclampsia and COVID‐19, such as the ACE‐2 receptor, which acts both as a binding site for the virus and as part of blood pressure control, or the inflammatory response and vascular activation common to both diseases. Previous studies showed a greater prevalence of pre‐eclampsia among COVID‐19 cases. A systematic review comparing pooled data from different settings reported a prevalence of pre‐eclampsia of 7.0% and 4.8% in cases with and without the infection, respectively, which corresponds to a pooled odds ratio of 1.62.^27^ In addition, maternal and perinatal outcomes were worse among cases of COVID‐19 plus pre‐eclampsia, compared with pre‐eclampsia only.^6^

During the pandemic, COVID‐19 surpassed other causes of maternal mortality and had a great impact in low‐ and middle‐income settings, especially before vaccination, highlighting delays within the healthcare system, referral, and management of emergencies.^28^ The present study represents a time frame in which the impact of COVID‐19 itself was smaller than the indirect consequences of the pandemic in the region, given the low number of confirmed infections among the study participants. In Brazil, the most severe period of the pandemic happened in 2021. As such, among pregnant women with confirmed COVID‐19 infection and severe acute respiratory syndrome, 5.9% died in 2020 and 11.8% died in 2021.^26^ A similar pattern was observed in the region of Campinas: 3.5% of pregnant women with COVID‐19 died in 2020, and, in 2021, 8.6% of them died.^26^

The delays in health care as a consequence of the pandemic are evidenced by data from the Brazilian Public Health System: there was a reduction of 65% in medical consultations and prenatal procedures by May 2020 nationally.^29^ Other countries, such as Kenya, suffered from a similar problem: women who gave birth during the pandemic delayed initiating prenatal care, especially due to difficulties in accessing healthcare services.^30^ It is well known that delays greatly impact maternal and perinatal outcomes,^31^ and previous Brazilian data regarding influenza H1N1 had already shown that any delays in health care were responsible for a two‐fold increase in severe maternal outcomes from 2009 to 2010.^32^ Delays in health care could explain the findings of less anti‐hypertensive therapy, reduction in diagnoses of early‐onset pre‐eclampsia, and later recognition of pre‐eclampsia with signs of severity for the first time. Considering pre‐eclampsia, early identification of risk factors, timely diagnosis, and referral for hospital surveillance are key to avoid adverse outcomes.^33^

This study also identified that the prevalence of LGA newborns varied in parallel with the prevalence of women with diabetes. Although it is not possible to determine a cause‐effect relationship between diabetes and LGA babies owing to the study design, the delays in health care might have impacted the adequate management of diabetes, thus resulting in large newborns, given the close relationship of these conditions in other contexts.^34^, ^35^

The COVID‐19 pandemic acted as a magnifying glass for identifying flaws in healthcare assistance globally. The maternity hospital considered should not be taken as representative of the country, because it is a well‐equipped referral center, with high‐quality ICU and neonatal ICU. This should account for the overall outcomes presented.

This study has limitations, including its retrospective design and reliance on medical chart reviews, and the limited number of COVID‐19 cases, preventing the assessment of the direct effect of the pandemic on the prevalence of pre‐eclampsia. Nevertheless, its broad assessment of cases allowed for understanding the impacts of the pandemic during pregnancy, hospital admission, and the postpartum period.

These findings support the need for policy‐makers to prioritize prenatal care counseling with timely and accurate identification of complications. During crisis, there needs to be special attention towards reproductive health, family planning, pregnancy, and postpartum care, with an effective surveillance system to report on outcomes.

In conclusion, the prevalence of pre‐eclampsia was overall around 10% and increased during the last considered pandemic period, at the institution studied. However, it is important to highlight that early‐onset diagnosis of pre‐eclampsia decreased, with a reduction in the frequency of prenatal antihypertensive therapy, most likely as a result of delays in health care and referral. The present data support the relevance of maintaining operational obstetric care even in times of crisis.

## AUTHOR CONTRIBUTIONS

JCS, JM, JPSG, and MLC contributed to conception and design of the work; JCS, VLVR, RRJ, MS, MAC, AJGO, LCV, and ABPR acquired, analyzed, and interpreted the data; and JCS, JM, JPSG, MLC, RTS, and JGC drafted and reviewed the manuscript. All authors are accountable for all aspects of this manuscript and vow for its accuracy.

## CONFLICT OF INTEREST STATEMENT

The authors have no conflicts of interest.

## Data Availability

Research data are not shared.
